# Book Review

**Published:** 1896-09

**Authors:** 


**Galen.** By James Finlayson, M.D. Pp. 55. Glasgow: Alex. Macdougall. 1895.—This is a most welcome pamphlet, enabling the reader to get in short space an ample grasp of the work of one who, as anatomist and practitioner, dominated medical life for centuries. By means of appropriate quotations and comments, the reason of this influence is well set forth by Dr. Finlayson, whose monograph consists of two bibliographical demonstrations given in the Library of the Faculty of Physicians and Surgeons of Glasgow. Every librarian who has facilities for doing so should follow Dr. Finlayson's excellent example.

**A History of the Chronic Degenerative Diseases of the Central Nervous System.** By Thomas Kirkpatrick Monro, M.D. Pp. vi., 82. Glasgow: Alex. Macdougall. 1895.—The title sufficiently indicates the scope of this work, which is reprinted with some slight alterations from the *Glasgow Medical Journal* for 1895. The labours of our predecessors in differentiating and defining diseases are too soon forgotten, and it is well that we should be reminded of the men to whose painstaking researches we owe so much. What Dr. Monro has done for the chronic degenerative diseases of the nervous system might well be done for other diseases. In reading the book one cannot but be struck with the fact that all real knowledge of these diseases has been the work of the last fifty years, and this affords a hopeful augury that the labour and attention now given to this subject by so many skilled workers will result in the accurate definition of many conditions which still remain obscure. The book is well written, and printed in excellent type. The author must be congratulated on having produced a work of much interest, especially to those engaged in the study of nerve diseases.

**Velada Fúnebre en Honra del Eminente Sabio Francés Luis Pasteur.** Pp. 48. Mexico. 1895.—The National Academy of Medicine of Mexico, always a great admirer of the genius of Pasteur, felt itself profoundly moved by the news of his death, and, to mark its sorrow at the sad event, adjourned the Session of the 2nd October, when the President of the Association officially announced his decease. The members of the Society, also desiring to make a public manifestation of their condolence and to honour the memory of this illustrious man, determined to celebrate a funeral vigil, the proceedings of which are recorded in this publication. So many wished to be present that the Chamber of Deputies was lent for the purpose. The President of the Republic, the Ministers of State, the French Consul and other foreign representatives, and the greater part of the men known to science and politics were invited to be present. The Chamber was draped with black and wreaths of laurel, and under a canopy formed by the flags of all nations was placed a large photograph of Pasteur. In the intervals of the addresses solemn music from Beethoven and Verdi was played by the band. The first oration was delivered by Dr. Angel Gaviño. It was of a most eloquent character, and was attentively listened to by the large assembly. He traced the history of this gifted scientist from his early days to the 27th of December, 1892, when he was presented with a gold medal by the Academy of Medicine, on which were engraved the words, "Science and Humanity are grateful to thee." His discoveries in lactic and alcoholic fermentation were described at length, and his investigations into the diseases of the silkworm, by which so large an industry of France was seriously affected; his researches in bacteriology, and, finally, his inoculations for hydrophobia were passed under review, and an impressive address was concluded with the words: "Gentlemen, to venerate such savants as he was is the fortunate privilege of civilised nations, and the example of his self-denial, constancy, and philanthropy will serve for generations to come as the model of the virtues which conduce to immortality." This was followed by the recitation of a poem specially composed for the occasion, and a speech to the same effect from Dr. Ramos.

**Index-Catalogue of the Library of the Surgeon-General's Office, United States Army.** Vol. XVI. W. — ZYTHUS. Washington: Government Printing Office. 1895.—Another volume of this eminently useful work has reached us. We have frequently expressed our admiration of it, and a further acquaintance fully justifies the high praise universally bestowed upon it. Some slight idea of the magnitude of the undertaking can be gathered from the fact that the mere titles of the periodicals used in the compilation of the work form a volume of 282 pages. We are glad to learn that a second series will be commenced at once.

**The Schott Treatment for Chronic Heart Diseases.** By RICHARD GREENE. Pp. 16. London: The Scientific Press, Limited. 1895.—In this little book Dr. Greene gives a lucid account of the treatment for heart-disease conducted at Nauheim. He adds his testimony to that of others that some cases of heart-disease markedly improve under its use, especially examples of simple dilatation without valvular disease.

**Practical Uranalysis and Urinary Diagnosis.** By Charles W. Purdy, M.D. Second Edition. Pp. xviii., 357. Philadelphia: The F. A. Davis Co. London: F. J. Rebman. 1895.—That a second edition of this book has been called for within a few months of the appearance of the first is sufficient evidence that it has supplied a want. Some further illustrations have been added and the text is said to have been carefully revised; otherwise it remains the same as in the first edition, which was recently reviewed in our columns.

**A Practical Treatise on Materia Medica and Therapeutics.** By John V. Shoemaker, M.D. Third Edition. Pp. ix., 1108. Philadelphia: The F. A. Davis Company. London: The F. J. Rebman Co., Ltd. 1895.—This work, formerly issued in two volumes, is now more conveniently compressed into one volume. Testing the information by reference to sundry headings, we judge that the work has been thoroughly and carefully brought up to date, and we can but reiterate the opinion expressed in noticing the first edition, that it is an encyclopædia of therapeutics in the widest sense of the term unrivalled in the English language.

**Rough Notes on Remedies.** By William Murray, M.D. Pp. 104. London: H. K. Lewis. 1896.—This small volume might well be described as the convictions of a sage observer in favour of the more active use of some well-known drugs. Fifteen minim doses of liq. arsenicalis, forty minims of tinct. belladonnæ, and eighty grains of calomel, are heroic doses, but their utility is abundantly demonstrated. The experienced practitioner has done his best to impart his methods for the guidance of others, who cannot fail to profit by a perusal of this much-needed record.

**The Natural Arsenical Waters of La Bourboule.** By A. M. Brown, M.D. Pp. 38. London: The Sanitary Publishing Company, Ltd. [n.d.]—The water of the Choussy-Perrière spring at La Bourboule is now well known, and its use does not of necessity imply a visit to the Auvergne, inasmuch as every care is taken in bottling the water for transport. Its range of utility is as wide as that of the principal constituent which it contains—viz., the arseniate of soda,—and a visit to the mountainous districts of Central France, with residence at an elevation of two to three thousand feet, may form a most agreeable and health-giving summer holiday.

**Health Notes for the Seaside: with special reference to Whitby and District.** By A. C. Dutt, M.B. Pp. 61. Whitby: Horne and Son. 1895.—The modest author of this little book has, during the intervals of private practice, compiled a series of practical and useful essays, of the common-sense type, ranging from the composition of dust up to the nature of the soul. They are well worthy of better type, of a more pretentious dress, and of more care in revision. An *édition de luxe* might well be provided by an enterprising publisher.

**The Pharmacopœia of the Evelina Hospital for Sick Children.** Second Edition. Pp. 53. London: J. & A. Churchill. 1896.—We notice a few additions in the new issue of this useful little book which make it more complete. There is no great originality in the prescriptions, but this one can hardly expect.

**A Pharmacopœia for Diseases of the Skin.** Edited by JAMES STARTIN. Fourth Edition. Pp. 53. Bristol: John Wright & Co. 1896.—No doubt there is some advantage in having a Pharmacopœia containing formulæ used in the treatment of skin affections, but we are very much surprised that a fourth edition (the cover calls it the third) of this work should have been required in its imperfect and somewhat crude condition. The forerunner of this little book is manifestly *The Pharmacopœia of the London Hospital for Diseases of the Skin*, published in 1848, the vague sentences and errors of which are here perpetuated. The classification of skin diseases is not up-to-date, and in the formulæ of remedies, as well as in the summary contained in the "Therapeutic Index," and especially under "Eczema," not a word is said about that very valuable remedy, salicylic acid. It is mentioned only—and that very casually—in the treatment of "Corns and Warts" and of "Pruritus."

**Dental Materia Medica and Therapeutics.** By JAMES STOCKEN. Fourth Edition. Revised by LESLIE M. STOCKEN and J. O. BUTCHER. Pp. 155. London: H. K. Lewis. 1895.—The new edition of this work is a most complete book of reference for all drugs that the dental surgeon may at any time want. As a matter of fact, it is the only book of the kind, as far as we know, which attempts to cull from a general Materia Medica the limited number of drugs which a dentist may wish to employ. It is well arranged and thoroughly well done.

**Public Health Laboratory Work.** By HENRY R. KENWOOD, M.B. Second Edition. Pp. xvi., 453. London: H. K. Lewis. 1896.—Since the issue of the first edition of this work, which was reviewed in this *Journal* in December, 1893, the opportunity has been taken of submitting it to a thorough revision; some processes given in the former edition have been replaced by others, and a considerable amount of new material has been added without increasing the size of the work. The student of public health will here find the essentials of laboratory work in the analysis of water, air, soil, and foods, conveniently arranged for practical work.

**The Prevention of Epidemics.** By FRANCIS VACHER. Pp. 16. London: W. H. & L. Collingridge.—This address, delivered at the annual meeting of the Incorporated Society of Medical Officers of Health in 1895, is interesting in that it contains conclusions, the outcome of several years' practical experience, upon many points of recurring interest in public health work.

**History of the Cholera Controversy.** By Sir GEORGE JOHNSON, M.D., F.R.S. Pp. xi., 78. London: J. & A. Churchill. 1896.—One of the latest of the many works of this vigorous and much lamented writer, this small volume puts forward a strong plea for the "eliminative and antiseptic" treatment of cholera, based upon an extensive experience dating from the epidemic of 1849. The last chapter contains practical directions for the treatment of cholera and of diarrhœa at epidemic periods.

**The Prophylactic Clothing of the Body.** By W. F. CLEVELAND, M.D. London: H. K. Lewis. 1896.—In this small pamphlet of fifteen pages the author devotes the first nine to a statement of the method of heat production and loss in the animal body and to quotations from Michael Foster and Herbert Spencer on this subject and on the effects of excessively cold climates in weeding out the feebler inhabitants. For two pages and a half the advisability of using flannel under-garments, hot-water bottles, socks at night, &c., is considered. The rest is taken up almost entirely with the causes of "chills" and how they act on the body: "A chill might predispose the system to imbibe the puerperal poison when it was, so to say, *afloat*, in the same way that diarrhœa, if neglected, predisposed the system to take the cholera bacillus, when it was lurking in the neighbourhood." This quotation may serve as a sample of the time-honoured but incorrect ideas which occasionally crop up in this address, although it is only fair to say that most of the precepts laid down in the few pages which deal with clothing are as sound as they are antiquated.

**A Manual of Medical Jurisprudence and Toxicology.** By HENRY C. CHAPMAN, M.D. Second Edition. Pp. 254. Philadelphia: W. B. Saunders. 1896.—This work remains essentially the same as the first edition, which we reviewed in 1893; but we note that there is an improvement in the type and in the paper. There are fifteen pages more of letter-press, as well as some additional woodcuts in the toxicological section.

**The Johns Hopkins Hospital Reports.** Volume IV. No. 9. Report in Pathology, IV. Baltimore: The Johns Hopkins Press. 1895.—Here Dr. Whitridge Williams reports a case of deciduoma malignum with the care and thoroughness that characterise the various branches of work connected with the Johns Hopkins Hospital. Dr. Williams considers the evidence afforded by microscopical examination throws doubts upon the view that these malignant growths commence in the decidua. The most striking feature of his case was the presence of finger-like processes of protoplasm containing several nuclei. These he calls masses of syncytium, the name given to the superficial layer of the chorionic villi. In other recorded cases of deciduoma malignum similar syncytial masses have been described. Dr. Williams considers that there can be no doubt that these masses are derived from the chorionic villi. If such a view be accepted, and it be also allowed that the opinion of the majority of authorities is correct, namely, that the cellular covering of the chorionic villi is maternal in origin, then the malignant growths known as deciduoma malignum are in reality uterine and not decidual.

**The Middlesex Hospital. Reports of the Medical, Surgical, and Pathological Registrars for 1894.** London: H. K. Lewis. 1895.—This vast and accurately detailed collection of facts forms a volume of 406 pages, and speaks well for the energies of registrars and pathologist. It comprises statistical tables of all the injuries and diseases for the year, with summaries of the notes on more important cases. It fully meets the possible exactions of a subscribing public, but fails to give the medical reader the full advantages of suitably selected arrangement, comment, and description. It contains an analysis of no less than seventy-three cases of enteric fever treated during the year with a mortality of 6.6 per cent. Sixty-five of the cases gave Ehrlich's reaction, and fifty-seven of them were treated by cold bathing or sponging. It is interesting to note that all the three medical patients with aneurysm had served in the army. It gives an abstract and notes of twenty-six operations on the kidney performed during the year, and of three cases of hydatid of liver or omentum. A case of perforating gastric ulcer was successfully operated on four hours after the perforation occurred. Here there was sudden pain at the onset; but the patient was brought to hospital without at first showing any collapse, disappearance of the liver dulness being the only early sign. There was a unique case of actinomycosis of the skin, very typical in its appearances. This is recorded in vol. lxxviii. of the *Transactions of the Royal Medical and Chirurgical Society of London*. Notes are also given, amongst others, of cases of typhus fever, hydatid of pleura, acute myelitis, Landry's paralysis, and splenectomy for simple hypertrophy. Although the reports are admirable in their completeness, they might be rendered much more valuable medically by suppression of unimportant details, and by the collective elaboration of important cases followed by the deduction of general principles.

**Saint Bartholomew's Hospital Reports.** Vol. XXXI. London: Smith, Elder, & Co. 1895.—St. Bartholomew's Hospital Reports will always be interesting, not only to past and present Bartholomew's men, but to the profession at large. Those who do not read the reports of our large hospitals often miss some of the most important papers published during the year. To Bartholomew's men—and indeed to the profession generally—the volume has a sad interest. It opens with an "In Memoriam" account of the life and character of Sir William Savory, a man familiar to all old students of the Hospital. Mr. Howard Marsh has shown himself to be an excellent biographer.

All sides of Sir William Savory's character and work are graphically represented, and those who knew him only in the wards and operating theatre, or perhaps only in the examination room at the College of Surgeons, will learn much to interest and please them. In the same year as the celebrated surgeon of St. Bartholomew's Hospital, the steward, Mark Morris, passed away: his anonymous biographer writes: "There is probably not a Bartholomew's man now living who did not know the Steward, and to whom the name will not recall one of the most familiar faces in the Hospital." To those who have held resident posts at the Hospital—who knew him well and appreciated him very highly—the short account of his life and long connection with the Hospital written by "the friendly hand of one whose memory goes back to the days when even Mark Morris was not yet an officer of the Hospital" cannot fail to be interesting. It would be impossible to refer to individual papers as of particular interest, so much depends on the interests of the reader. Dr. Hepburn's paper on "Mal des Montagnes; or, so-called Mountain-Sickness," is a very thorough and valuable contribution to our knowledge of this little-known and less understood disease. The psychologist will find ample material for study in Dr. Claye Shaw's paper "On Cell-Memory." Surgeons will be interested in a joint paper by Mr. Butlin and Mr. Colby "On Sarcoma of the Bones of the Thigh and Leg." "The Prognosis of Tetanus," by Dr. Worthington is of particular value in connection with the antitoxin treatment. Diphtheria is the subject of two communications—one from Dr. Herringham on the treatment by antitoxin at St. Bartholomew's Hospital, and the other a bacteriological investigation in this disease, by Dr. Kanthack and Mr. White. "A Case of Actinomycosis of the Thoracic Wall" is of great interest both to the physician and surgeon. It is reported by Sir Dyce Duckworth. Anæsthetists will be glad to find a paper "On the Mechanical Factor in Chloroform-Anæsthesia," by Mr. Gill, the Senior Anæsthetist to the Hospital.

**St. Thomas's Hospital Reports.** New Series. Vol. XXIII. London: J. & A. Churchill. 1896.—The volume begins with a warm tribute to the memory of the late Dr. Bristowe. Of the many eminent men on the roll of physicians of St. Thomas's Hospital, he was one of the ablest and most accomplished. It is hardly necessary to say here, for the number and value of his contributions to medicine and pathology sufficiently attest it, that Dr. Bristowe was unsurpassed in the extent of his professional knowledge. Less generally known perhaps, as lying apart from his professional reputation, were his high artistic and literary gifts. As a clinical teacher he was amongst the very first, and many generations of students look back with gratitude to the training in medicine which they owe to him. He inspired them moreover with that deep respect and affection which were only due to his nobleness of character and unvarying kindness of heart.

An excellent portrait of Dr. Bristowe forms the frontispiece to the volume. Dr. Payne has an interesting paper on some anatomical and practical observations of James Molins, a surgical pupil and apprentice at the hospital from 1674—1677. We notice amongst other papers "A Note on the Physiology of the Spinal Cord," by Professor Sherrington, an account of two interesting cases of severe injury to the head by Mr. W. M. Battle, and a well-marked example of intra-uterine rickets by Mr. Makins. Dr. Theodore Acland and Mr. Chas. Ballance contribute a very important and valuable monograph on the subject of cerebellar abscess secondary to ear disease. This contains the report of a case successfully operated on, and of great interest from the point of view of the symptoms present. They describe the character and mode of causation of the symptoms in cases of cerebellar abscess, give abstracts of 77 cases collected from the literature of the subject, and statistics of chief symptoms in 100 cases together with an extensive bibliography, and a full discussion of the differential diagnosis. The paper is very well written, and deserves careful study. There are the usual reports of the work carried on in the medical, surgical, and special departments of the hospital, with short abstracts of cases of special importance and interest. These show the amount of good work that has been done at the hospital during the year.

**Recherches cliniques et thérapeutiques sur l'Épilepsie, l'Hystérie et l'Idiotie.** Volume XV. Paris: Félix Alcan. 1895.—This is the annual report of the department of the Bicêtre Hospital devoted to idiots, under the charge of M. Bourneville, and gives the usual statistics. In addition, the second part contains a full account of the methods of observation and examination which are carried out. This is published in refutation of an attack made upon M. Bourneville with respect to his methods of physical examination. The third part gives a full report, with necropsy, of a case of congenital idiocy with paraplegia, illustrated by three beautiful plates of the morbid changes in the brain, and of a case of myxœdematous idiocy (sporadic cretinism).

**Transactions of the Association of American Physicians.** Vol. X. Philadelphia: Printed for the Association. 1895.—This working society of one hundred members, all of whom have done good work, having no regard for medical politics or for medical ethics, here issues its tenth volume of noteworthy contributions to scientific and practical medicine, containing very much of the cream of American medical literature. Dr. Jacobi's record-breaking case of hyperthermy, in which, in spite of scepticism and every possible precaution, thirty-four observations in the course of five days gave an average temperature of 124° or 125° (the highest being 148°, the lowest 109.8°), is given in carefully-described detail. Dr. Whittaker, in his paper on the etiology of idiopathic hypertrophy of the heart, gives syphilis its correct position as ranking next to alcohol in the production of cicatricial myocarditis.

**Transactions of the American Surgical Association.** Vol. XIII. Philadelphia: William J. Dornan. 1895.—This volume is full of interest and will be welcomed by all surgeons on this side of the Atlantic. Professor William White, of Philadelphia, contributes a long and important article on "The Results of Double Castration in Hypertrophy of the Prostate," in which he brings forward abundant additional proofs of the safety and efficiency of this method of treating the disease. He replies to various objections which have been made to his operation, and tabulates 111 cases with only 20 deaths. Important papers are contributed on operative treatment of cancer in various localities, including "The Female Generative Organs," by Dr. John Homans; "The Lip, Tongue, Floor of Mouth, and Pharynx," by Dr. P. S. Conner; "Treatment of Cancer of the Breast," by Dr. J. S. Wight; "Operative Treatment of Cancer of the Male Genitals," by Dr. Hunter McGuire; and "The Modern Operative Treatment of Rectal Cancer," by Dr. Arpad G. Gerster. All of these papers are accompanied by a full report of the discussions which followed. The editing of this volume is not carried out in the customary careful manner, for we notice many errors in the spelling of proper names, such as Fauls for Faulds (p. 130), Monroe Smith for Munro Smith (p. 287), MacCormick for MacCormac (p. 285), Mounsell for Maunsell (p. 84), Lowrie for Lawrie (p. 179), and Symond for Symonds (p. 185).

**Transactions of the College of Physicians of Philadelphia.** Third Series. Vol. XVII. Printed for the College. 1895.—With the 171 pages of papers on a variety of subjects is bound up the Jenks Prize Essay on "Infantile Mortality during Child-birth and its Prevention," by Dr. A. Brothers. The author observes that "since the recent results of the Cesarian section, and of the revived symphyseotomy, have assumed so favorable an aspect, we are on the threshold of a new era in the development of the obstetric art," and as a result of ten years' active midwifery practice, combined with a large experience in the fields of pediatrics and everyday gynecology, "he feels that his points of observation have been sufficiently comprehensive to allow him to carefully weigh the subject in both hands." He has endeavoured to point out the advances made in recent years in the interests of the unborn child previous to labour, during the critical hours of actual labour, and in the earliest period of life succeeding labour.

**Transactions of the Colorado State Medical Society.** Denver: A. J. Ludditt. 1895.—This volume of 551 pages contains by-laws, list of members, and a series of photographs of past presidents, the originals of which must have been quite ornamental to the State. Dr. Charles S. Manly, in his paper on "The Importance of Rational Methods in the Diagnosis of Phthisis with special Reference to pre-Tuberculosis," lays down the axiom that all wasting diseases should be suspected to have a tubercular origin and tendency. Dr. Charles Denison gives a report on the results of treatment by antiphthisin (Klebs).

**Transactions of the Medical Society of Virginia.** Richmond: J. W. Fergusson & Son. 1895.—A noteworthy speciality of this volume is that all the Fellows of the Medical Society of Virginia are requested to supply the Secretary with a correct biographical register in order that the Necrological Committee may be in readiness to provide a suitable memoir of their departed friends. This method of getting each member to write an autobiography is one which, if generally adopted, would save much trouble when a biographical notice is required.

**Transactions of the New Hampshire Medical Society.** Concord: The Republican Press Association. 1895.—The 186 pages of this record contain the usual presidential address, with dissertations on "The Physical Basis of Crime," "Cancer," "Germ Diseases," and many other topics, treated in an original and interesting manner. The volume should have an index.

**Transactions of the American Ophthalmological Society.** Vol. VII., Part II. Hartford: Published by the Society. 1896.—This volume contains thirty-five communications, some of which are very valuable contributions to ophthalmology. Dr. Charles Stedman Bull in a most careful report of 612 cases of squint for which he had operated, found that 94 per cent. had some form of hypermetropia. His paper shows the inadvisability of simultaneous tenotomy of both internal recti in all but a few exceptional cases. Divergence followed in 6 cases out of 20 so dealt with. Neurologists will read with interest an account of a case of recurrent oculo-motor palsy associated with neuralgia, which is interesting in connection with the series of similar cases recently collected by Dr. Ormerod and Mr. Holmes Spicer. Dr. Risley relates a case of what Cohen has described as vaso-motor ataxia with monocular exophthalmos. The difficult subject of the astigmatic effect of spherical lenses when placed obliquely to the axis of vision is admirably treated by Dr. John Green in a paper going fully into the optics of the conditions involved. It explains the well-known but little understood advantages that patients often find from tilting their spectacles into "incorrect" positions. In the statistics of 4,000 cases of ocular headaches, for which Dr. Mittendorf had ordered glasses, we are surprised to see that no less than forty per cent. had less than 0.5 D simple hypermetropic astigmatism. But what is equally surprising is that only 6½ per cent. had compound hypermetropic astigmatism. In estimating the refraction no mention is made of any mydriatic being used with a view to detecting latent hypermetropia, or of any objective test being used to check the results. His statistics, therefore, are of but little value, and we are not astonished to find that of late he has frequently ordered 0.125 D cylinder (25 feet focal length roughly), with which he says he has seen satisfactory results. Dr. W. F. Norris, in a paper on The Terminations of the Rods and Cones in the Retina, describes them as sometimes ending in loops, but the six micro-photographs which accompany his paper do not at all support his contention. Dr. Albert Heyl relates and illustrates two interesting and rare ophthalmoscopic cases. One is a spontaneous rupture of the choroid running radially through the macular region, which he attributes (on what grounds is not stated) to sudden spasmodic contraction of the external rectus muscle. The other is a case of what Dr. Heyl takes to be albuminoid deposit in the discs and retinæ of both eyes, but not materially affecting vision. Dr. G. E. de Schweinitz reports another case of traumatic exophthalmos coming on within four days of an injury to the head, but without any evidence of fracture of the orbit. In a discussion on the needling of opaque capsules, it appears that the well-known and useful method with two needles, proposed by Sir William Bowman more than forty years ago, is rarely resorted to in America. Dr. Sutphen advocates the use of salicylate of soda in glaucoma, and, from the accounts he gives of six cases, it certainly seems worth trial where for any reason operation is undesirable. Dr. Noyes relates five cases of severe hemorrhage after extraction of cataract. He thinks this "tragic" occurrence, as he aptly calls it, is so likely to happen after operation on myopic eyes in old people that he would in many cases entirely abandon the idea of operating.

**Transactions of the Medical Society of London.** Vol. XVIII. London: Harrison and Sons. 1895.—The papers here reproduced have, for the most part, appeared in full in the weekly journals, and are familiar to the most superficial medical reader. The Lettsomian lectures of Dr. Frederick T. Roberts require careful reading, and remind one of the system adopted in the author's well-known text-book. The paper on "Cycling and Heart Disease" by Sir Benjamin Ward Richardson is of especial interest just now, when so many ladies are leaving their horses to be exercised by the groom, whilst they themselves are indulging in more health-giving exercise on cycles: the last word on this subject is worthy of being quoted: "Discarding overstrain, he saw no reason to doubt that cycling was very beneficial to women, especially those with a tendency to anæmia." If any direct or obvious harm is likely to arise in healthy women, there will soon be ample opportunity of obtaining much evidence on the subject; but, on the other hand, it appears reasonable to suggest that in cases of weak circulation both in men and women the use of the cycle may well be adopted instead of the elaborate and tedious methods associated with the name of the brothers Schott at Nauheim.

**Transactions of the Clinical Society of London.** Vol. XXVIII. London: Longmans, Green, and Co. 1895.—As usual this volume of Transactions contains a large number of valuable papers and reports of cases. Where all are good it would be invidious to single out particular papers, and it is sufficient to say that the present volume fully maintains the high standard of its predecessors.

**Burdett's Hospitals and Charities.** 1896. By Henry C. Burdett. London: The Scientific Press (Limited).—Mr. Burdett's "annual," if a little late, is none the less welcome. It is a book which is indispensable to anyone interested or engaged in charitable work. A new feature in this year's publication is a table giving the income and expenditure of some of the great charities in the United Kingdom. Mr. Burdett does not despair of making in the future a complete return, but, incomplete as the list is, the labour is well repaid in the value of the table. Chapter II. we specially recommend as worthy of careful study. Its title, "The Misuse of Hospitals," shows that it treats of a subject which is occupying the minds of medical men very largely at the present time. We do not agree with all Mr. Burdett says, more particularly as to the reduction of cost per bed by payment of the staff (pp. 98—100), which would put "everything connected with the hospitals . . . upon a business basis." It certainly does not seem probable in the present impoverished state of the medical charities that such a Utopian plan will be carried out. Among other things, the poor Radcliffe Infirmary comes in for its usual castigation, and "Dr." Barnardo's accounts are published *in extenso*, to show "how *not* to do it."

**Year-Book of the Scientific and Learned Societies of Great Britain and Ireland.** London: Charles Griffin and Company, Limited. 1896.—All who know this work agree that it is a most useful book of reference; it is well printed and appears to be generally correct, but the report of our society shows that no "proof" had been submitted to the Secretary. The publication would be greatly improved if the index included the titles of the papers and the names of their authors.

**A Short History of Bookbinding.** Pp. 28. London: Printed at the Chiswick Press. 1895.—This is one of the most unobtrusive and pleasing forms of advertisement we have seen. The "short history," condensed from Mr. Zaehnsdorf's larger work on the subject, is useful and interesting, and, together with a glossary and a dainty frontispiece, makes the booklet very acceptable. The Zaehnsdorf work is too well known to need any commendation from us.

**Complete Catalogue of the Products of the Laboratories of Parke, Davis & Co., Detroit.** British Edition. 1896.—This forms a compact and convenient volume of 228 pages, containing much valuable information on drugs and foods for invalids. Many of these deserve a more extended use than they have yet received, but all good things have to earn a reputation by slow and steady progress.

